# The 5′-untranslated region of p16^INK4a^ melanoma tumor suppressor acts as a cellular IRES, controlling mRNA translation under hypoxia through YBX1 binding

**DOI:** 10.18632/oncotarget.5387

**Published:** 2015-10-17

**Authors:** Alessandra Bisio, Elisa Latorre, Virginia Andreotti, Brigitte Bressac-de Paillerets, Mark Harland, Giovanna Bianchi Scarra, Paola Ghiorzo, Robert C. Spitale, Alessandro Provenzani, Alberto Inga

**Affiliations:** ^1^ Laboratory of Transcriptional Networks, Centre for Integrative Biology, CIBIO, University of Trento, Trento, Italy; ^2^ Laboratory of Genomic Screening, Centre for Integrative Biology, CIBIO, University of Trento, Trento, Italy; ^3^ Laboratory of Genetics of Rare Hereditary Cancers, DiMI, University of Genoa, Italy and IRCCS AOU San Martino-IST, Genoa, Italy; ^4^ Service de Génétique, Institut Gustave Roussy, Villejuif, France; ^5^ Section of Epidemiology and Biostatistics, Leeds Institute of Cancer and Pathology, University of Leeds, Leeds, UK; ^6^ Department of Pharmaceutical Sciences, University of California, Irvine, CA, USA

**Keywords:** p16^INK4a^, YBX1, melanoma, IRES, hypoxia

## Abstract

CDKN2A/p16^INK4a^ is an essential tumor suppressor gene that controls cell cycle progression and replicative senescence. It is also the main melanoma susceptibility gene. Here we report that p16^INK4a^ 5′UTR mRNA acts as a cellular Internal Ribosome Entry Site (IRES). The potential for p16^INK4a^ 5′UTR to drive cap-independent translation was evaluated by dual-luciferase assays using bicistronic and monocistronic vectors. Results of reporters' relative activities coupled to control analyses for actual bicistronic mRNA transcription, indicated that the wild type p16^INK4a^ 5′UTR could stimulate cap-independent translation. Notably, hypoxic stress and the treatment with mTOR inhibitors enhanced the translation-stimulating property of p16^INK4a^ 5′UTR. RNA immunoprecipitation performed in melanoma-derived SK-Mel-28 and in a patient-derived lymphoblastoid cell line indicated that YBX1 can bind the wild type p16^INK4a^ mRNA increasing its translation efficiency, particularly during hypoxic stress. Modulation of YBX1 expression further supported its involvement in cap-independent translation of the wild type p16^INK4a^ but not a c.-42T>A variant. RNA SHAPE assays revealed local flexibility changes for the c.-42T>A variant at the predicted YBX1 binding site region. Our results indicate that p16^INK4a^ 5′UTR contains a cellular IRES that can enhance mRNA translation efficiency, in part through YBX1.

## INTRODUCTION

Cap-dependent ribosome recruitment to mRNAs is the prevalent translation initiation process [1, 2] but cells are able to shift to a cap-independent mechanism in response to specific environmental conditions and cellular stresses [3–5]. Herein, translation is initiated through ribosome subunits' loading at a more internal site in 5′UTR region of mRNAs, the so-called Internal Ribosomal Entry Sites (IRES). IRES-driven translation initiation is generally favored in stress conditions such as inflammation [6], hypoxia [7] or amino acids starvation [8] when cap-dependent translation is reduced [9, 10] [11]. IRES-driven translation has been demonstrated for many important cancer genes, the majority being oncogenes, *e.g*. Vascular Endothelial Growth Factor (VEGF) [12, 13] c-MYC [14, 15], BCL2 [16], and XIAP [17], but with notable examples in the tumor suppressor class with the TP53 and p27 genes [18–20].

Melanoma develops from the malignant transformation of melanocytes which are localized at the dermal-epidermal junction, in physiologic hypoxic niches [21]. *CDKN2A/p16^INK4a^* is the main melanoma susceptibility gene identified to date [22–24]. CDKN2A/p16^INK4a^ (herein indicated as p16^INK4a^) is a critical tumor suppressor that inhibits the cyclin-dependent kinases CDK4 and CDK6, thereby keeping the retinoblastoma protein (pRB) in a hypo-phosphorylated state, thus leading to G1/S checkpoint activation [25]. Hypoxia leads to an increase of p16 level in epithelial cells, supporting its role in hypoxia-induced growth inhibition [26]. In addition, p16^INK4a^ plays a crucial function in the process of replicative senescence [27].

Given its critical role in cell homeostasis, there is much interest in understanding the molecular regulators of p16^INK4a^ expression. Since we have previously demonstrated that a few sequence variants in the p16^INK4a^ 5′UTR found in melanoma patients can have a negative functional impact, potentially acting at post-transcriptional level [28], we hypothesized that p16^INK4a^ 5′UTR might contain specific sequence and structural features that can drive cap-independent translation.

Here we demonstrate that p16^INK4a^ 5′UTR acts as a cellular IRES and we discovered YBX1 as a positive regulator of p16^INK4a^ cap-independent translation under hypoxic stress both in cancer-derived cell lines and p16^INK4a^ wild type lymphoblastoid cells obtained from a melanoma patient. Y-box binding protein 1 (YBX1) is a member of the CSD (cold-shock domain) protein superfamily over-expressed in several types of cancer including melanoma [29]. YBX1 can act as a transcription regulator but it is also able to regulate mRNA translation acting as ITAF (IRES Trans Acting Factor) for some mRNAs, such as c-MYC [30]. We demonstrate that a germline sequence variant found in the p16^INK4a^ 5′UTR (c.-42T>A) of a multiple primary melanoma patient results in local flexibility changes in RNA structure, impairing the binding of YBX1 and its stimulatory effect on IRES-dependent translation efficiency. This sequence variant appears to alter p16 protein expression. Impaired p16 translation under hypoxia could provide a mechanistic clue to explain melanomagenesis associated with this germline variant.

## RESULTS

### The p16^INK4a^ 5′UTR mediates cap-independent translation

**Figure 1:**
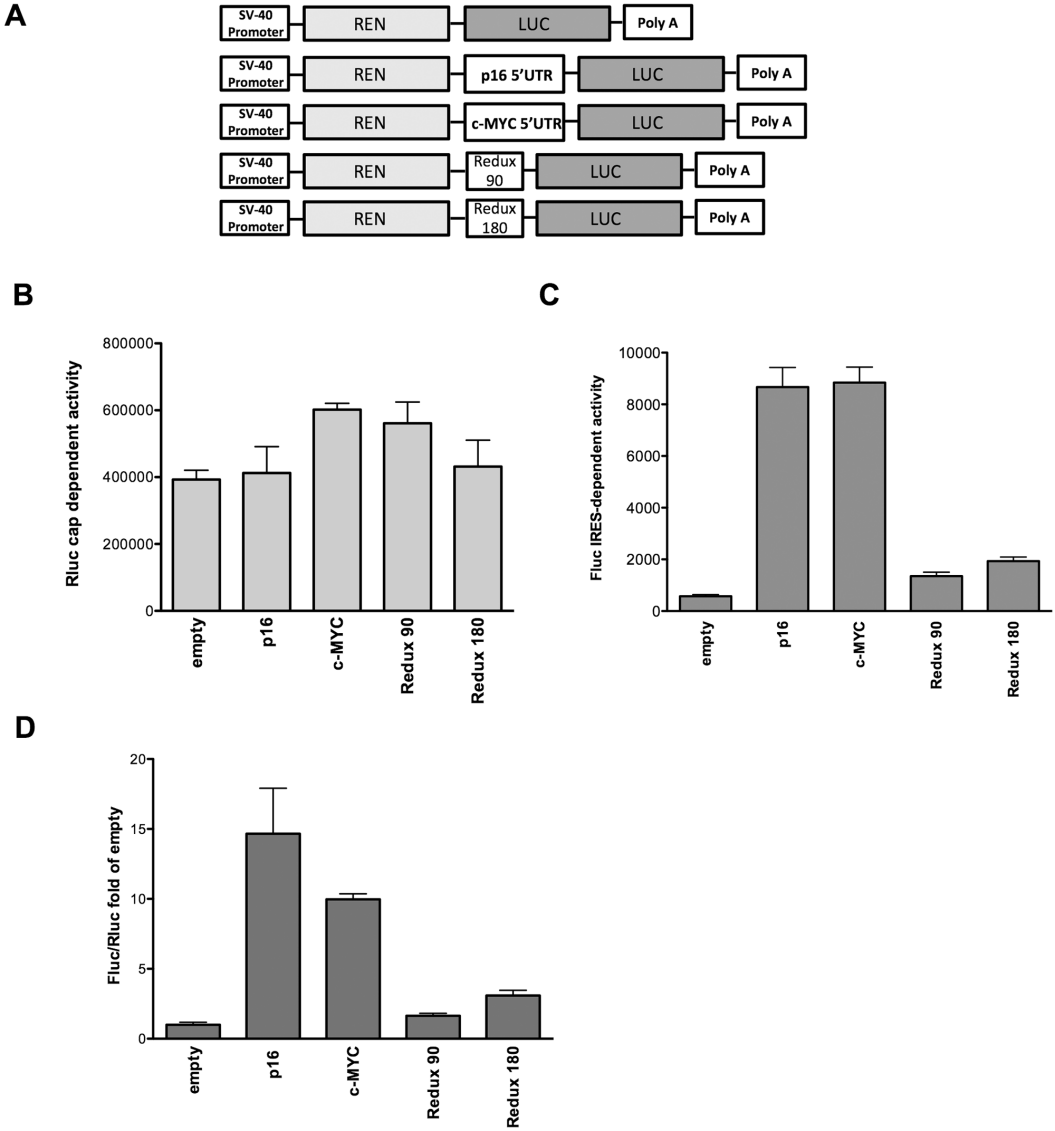
The p16^INK4a^ 5′UTR mediates cap-independent translation **A.** Schematic representation of the different pRuF-based bicistronic reporter vectors containing Renilla luciferase cDNA (light gray) under the control of the constitutive promoter pSV40 and p16^INK4a^ 5′UTR (full-length and two deletion fragments lacking, respectively, the proximal 90 nucleotides – Redux 180- or the distal 180 nucleotides –Redux 90-) placed upstream of the Firefly luciferase cDNA (dark gray). In these vectors Renilla luciferase protein (Rluc) is translated in a cap-dependent manner, while Firefly luciferase protein (Fluc) synthesis can be enhanced through a cap-independent mechanism. The fragment containing the c-MYC 5′UTR IRES site was used as a positive control. The pRuF-empty vector was used as negative control. **B, C.** Luciferase assays performed in MCF7 cells transiently transfected with the different pRuF reporter constructs. Results are shown as Renilla (Rluc – B) and Firefly luciferase (Fluc – C) raw data. Bars represent averages and standard deviations of at least three independent biological replicates. **D.** Data obtained from B and C panels were analyzed as ratio between Fluc and Rluc (Relative Light Units, RLU) and then normalized with the results obtained with a pRuF-empty vector (Fluc/Rluc fold of empty, where the empty value is set to 1).

A panel of bicistronic reporters where the full-length p16^INK4a^ 5′UTR or two different deletion fragments cloned as intervening sequences between Renilla and Firefly luciferase genes were used for transient transfection assays in MCF7 cells (Figure 1A). The portion of c-MYC 5′UTR described to exhibit a cellular IRES activity [31] was used as positive control (Figure 1A). Although Rluc levels were rather similar among the different samples (Figure 1B), the p16^INK4a^ and c-MYC 5′UTRs led to a significant increase in Fluc activity compared to the empty vector, indicating putative cap-independent translation of the reporter protein (Figure 1C). The deletion of the proximal 90 nucleotides (Redux 180) or of the more distal 180 nucleotides (Redux 90) of the p16^INK4a^ 5′UTR relative to the Fluc AUG site led to a remarkable reduction in luciferase activity, indicating that the entire 5′UTR sequence is necessary for Fluc activity. Results from Figure 1B and 1C were further analyzed as ratios between Fluc and Rluc measurements and presented as fold of induction compared to the pRuF-empty vector (Figure 1D). Additional controls excluded the possibility that the observed increases in Fluc activity were due to the presence of an alternative splicing event or of a cryptic promoter activity within the p16^INK4a^ 5′UTR (Figure S1). From these results we propose that the p16^INK4a^ 5′UTR can act as a cellular IRES, at least when ectopically placed in a bicistronic reporter construct.

### Hypoxia and mTOR inhibitors enhance p16^INK4a^ 5′UTR-mediated translation

**Figure 2:**
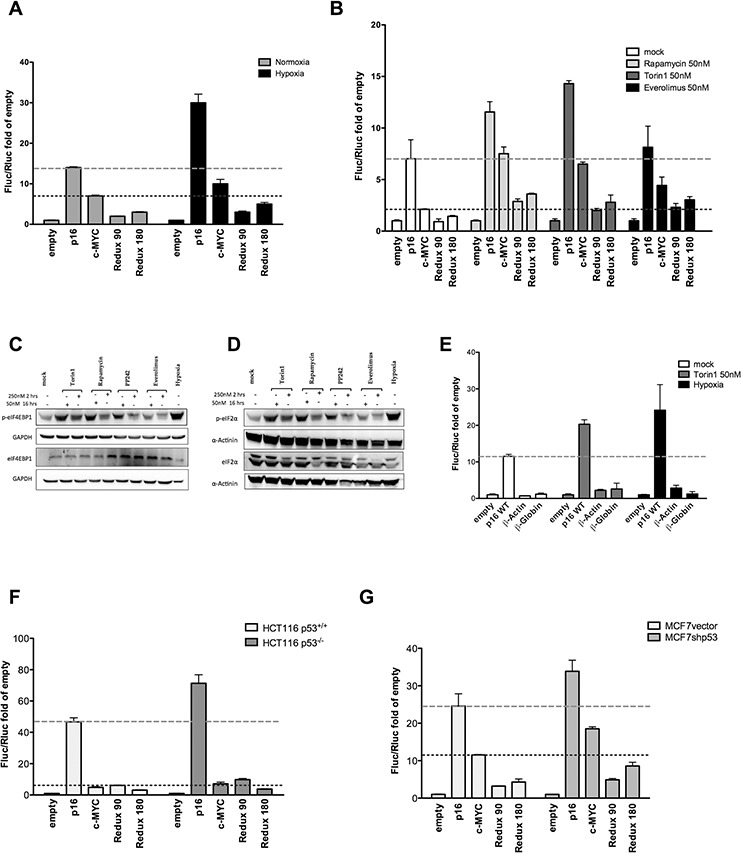
Hypoxia and different mTOR inhibitors enhance p16^INK4a^ 5′UTR mediated translation MCF7 cells transiently transfected with the different pRuF reporter constructs were grown in normoxic or severe hypoxic conditions **A.** or treated with mTOR inhibitors (Rapamycin, Torin1, or Everolimus) **B.** each at 50nM for 16 hours. Luciferase assays were performed as described in Materials and Methods and presented as described for Figure 1D. The individual Firefly and Renilla reporters' values are shown in Figure S2A and S2B. **C, D.** Western blot analysis showing the effect of mTOR inhibition and hypoxia treatments on eIF4E-BP1 phosphorylation status C. or on eIF2α phosphorylation D. MCF7 cells were treated with two different concentrations of the indicated drugs (250nM for 2 hours or 50nM for 16 hours). The expression of total eIF4E-BP1 or total eIF2α were used as controls. GAPDH or α-Actinin endogenous levels served as reference proteins. **E.** The impact of mTOR inhibition (analyzed with the two most effective treatments, 50nM Torin1 and hypoxia) was evaluated on the p16^INK4a^ 5′UTR sequence and two pRuF constructs containing the β-Actin and β-Globin 5′UTRs negative controls. The individual Firefly and Renilla reporters' values are shown in Figure S2C. **F, G.** Luciferase assays were also conducted using the same reporter constructs in HCT116 p53^+/+^ and p53^−/−^ cells F. or in MCF7vector and MCF7shp53 G. Dashed lines mark the activity of the p16 or c-MYC constructs in the mock condition, to better highlight the impact of the various treatments.

To further study the regulation of p16^INK4a^ 5′UTR, MCF7 cells were transiently transfected with pRuF vectors and then cultured in conditions where cap-dependent translation was inhibited. Hypoxia strongly stimulated the IRES activity of p16^INK4a^ 5′UTR also in comparison with the c-MYC 5′UTR. The p16^INK4a^ deletions led to only a slight increase in relative Fluc activity (Figure 2A).

Next, we used three different mTOR inhibitors to inhibit cap-dependent translation. Rapamycin and Torin1 treatment led to higher relative activity of the p16^INK4a^ as well as the c-MYC IRES-dependent Fluc reporter, while the effect of Everolimus was visible only for the c-MYC construct (Figure 2B). Western blot analysis was performed to detect the phosphorylated form of eIF4E-BP1, a downstream target of the kinase activity of mTOR, and of the translation initiation factor eIF2α. Given that these drugs can inhibit cap-dependent translation (mainly via mTOR inhibition) after two hours of treatment when given at a 250nM dose [32], we included this condition for comparison. The catalytic mTOR inhibitor PP242 was also included as a control. PP242 did not reduce the phosphorylation of eIF4E-BP1 at our experimental time point, but resulted in higher levels of the inhibitory phosphorylation of eIF2α (Figure 2C, 2D). Next we compared the impact on Fluc reporter activity of the wild type p16^INK4a^ 5′UTR with that of the β-actin and β-globin 5′UTRs, considered as negative controls for IRES-like activity [33]. Torin1 treatment and hypoxia were compared (Figure 2E). Unlike p16^INK4a^, the β-actin and β-globin 5′UTRs vectors were nearly identical to the empty pRuF vector.

Since it has been demonstrated that p53, through transcriptional repression of Fibrillarin (FBL), is able to negatively impact on IRES-dependent translation [34], we performed luciferase assays in HCT116 p53^+/+^, their derivative p53^−/−^ [35] as well as MCF7vector and their derivative MCF7shp53 [36] cells. The results indicated that in the p53 null or knock-down cell line, the relative activity of the Firefly reporter downstream of the p16^INK4a^ 5′UTR IRES was enhanced (Figure 2F, 2G). Relative expression of the FBL gene was indeed higher for the p53 proficient cells compared to the matched p53 deficient clones (Figure S1D).

### Endogenous p16^INK4a^ mRNA exhibits higher translation potential under conditions of reduced global protein synthesis

**Figure 3:**
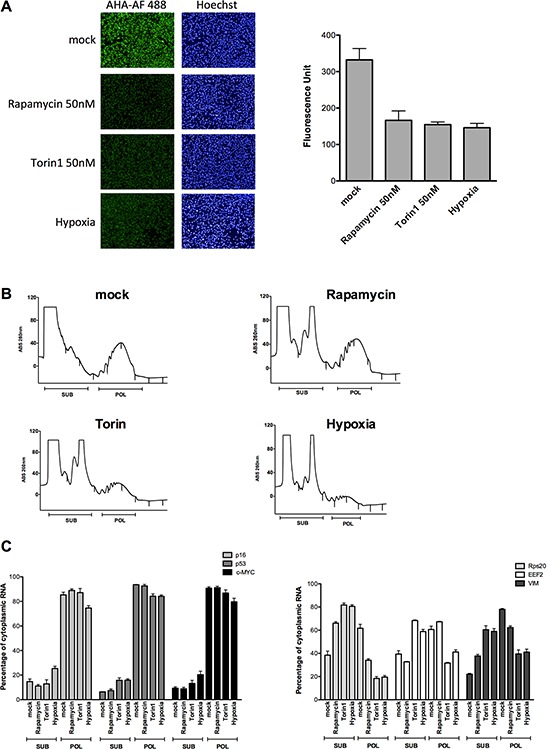
p16 mRNA retains polysomal loading in conditions of global translation inhibition caused by hypoxia or mTOR inhibitors **A.** SK-Mel-28 cells were cultured in hypoxia or treated with mTOR inhibitors (50 nM Rapamycin or Torin1) for 16 hours. The effect on global translation was quantified by imaging the relative incorporation of the methionine analog (AHA) using the Operetta High Content Imaging System (see also Supplementary Information). Nuclei were stained using the fluorescent dye Hoechst (left panels). On the right panel a quantification of the Alexa-Fluor 488 signal is presented. Bars represent averages and standard deviations of four biological replicates. **B.** Cytoplasmic lysates of SK-Mel-28 cells treated as for the experiment of panel A were subjected to sucrose-gradient ultracentrifugation. Polysomal profiles obtained after the various treatments are shown. Fractions corresponding to subpolysomes (40S, 60S and 80S) and polysomes (two or more ribosomes) were pooled together for RNA extractions as indicated. **C.** Relative quantification by qPCR of the p16, p53, c-MYC (left panels), Rps20, EEF2 and VIM (right panel) mRNAs. Bars plot the average percentage of cytoplasmic mRNA present in subpolysomal or polysomal fractions. The standard deviation of two biological replicates and a total of six technical replicates are shown. Results were normalized for RNA extraction and retrotranscription efficiency using a spike-in synthetic mRNA (see Methods). The total amount of recovered mRNAs from subpolysomal and polysomal fractions for each condition was measured to present the data as percentage of cytoplasmic mRNA. For the genes in the right panel, the treatments led to an increase in the proportion of the mRNAs in subpolysomal fractions compared to the polysomal fractions, with the exception of Rapamycin for EEF2. This pattern was not observed for p16, p53 and c-MYC mRNAs, indicating that their association with the polysomes does not change even when cells are reducing translation (see panel A).

To begin exploring the translation potential of the endogenous p16^INK4a^ mRNA we exploited polysomal profiling coupled to quantitative PCR in melanoma-derived SK-Mel-28 cells expressing p16 [28, 37]. First, we verified that the treatments with mTOR inhibitors (Rapamycin and Torin1 at the 50nM dose) or the culture in hypoxia led to a reduction in global mRNA translation (Figure 3A). Polysomal profiles (see Methods) showed an increase in the 80S peak with the different treatments and the polysomal fraction was particularly reduced after Torin 1 treatment or in hypoxia (Figure 3B). RNA was recovered from mock or treated cells and the relative percentage of cytoplasmic mRNAs associated with subpolysomal and polysomal fractions was quantified for p16, c-MYC and p53 mRNAs for which cap-independent translation potential has been reported [14, 18], and for Rps20, EEF2 and VIM that as TOP or TOP-like mRNAs are strongly dependent on mTOR activity for their translation [38]. qPCR revealed that while the treatments did not change polysomal association of p16, p53 and c-MYC, a reduction was visible for the TOP or TOP-like mRNAs particularly after Torin 1 treatment or hypoxia (Figure 3C). Considering polysomal association as an approximation of translation efficiency, these results strongly suggest that the p16 mRNA exhibit higher translation efficiency in conditions where global mRNA translation is reduced.

### Endogenous p16^INK4a^ protein expression is regulated by a post-transcriptional mechanism

**Figure 4:**
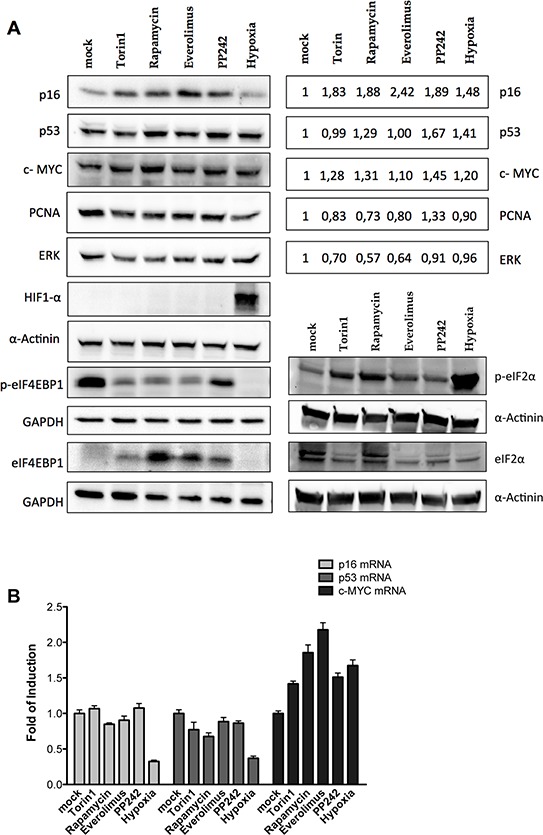
Endogenous p16^INK4a^ is controlled via a post-transcriptional mechanism in melanoma cells **A.** Western blot analysis were performed in the SK-Mel-28 melanoma-derived cell line treated with mTOR inhibitors (Rapamycin, Torin1, Everolimus and PP242, each at 50 nM) or grown in severe hypoxia, for 16 hours. Nitrocellulose membranes were probed with primary antibodies against the endogenous p16^INK4a^, mutant p53, c-MYC, PCNA, ERK1, HIF1α, phospho-eIF4E-BP1, eIF4E-BP1, phospho-eIF2α and eIF2α proteins. GAPDH and α-Actinin served as reference proteins. Quantification of p16, mutant p53, c-MYC, PCNA and ERK1 proteins (normalized to α-Actinin reference protein) are presented in the boxes on the right as average of the results obtained in two biological replicates. **B.** qPCR was conducted on total RNA extracted from SK-Mel-28 cells treated as shown in panel A. B2M and GAPDH were used as reference genes to normalize data. Bars represent averages and standard deviations of at least three independent biological replicates.

Using protein extracts from SK-Mel-28 we observed an increase in p16^INK4a^ protein levels upon treatment with the mTOR inhibitors, but not in hypoxic culture conditions (Figure 4A). We also assessed the endogenous levels of p53 and c-MYC. As expected, p53 and c-MYC protein levels were increased (Figure 4A). As negative controls we used PCNA (Proliferative Cell Nuclear Antigen) and ERK (Extracellular Regulated Kinase 1) which have no reported evidence of IRES activity in their 5′UTR. PCNA and ERK1 protein levels did not change or were even lower compared to the untreated cells. HIF1α protein stabilization confirmed that hypoxia was achieved in the culture conditions. The phosphorylation status of eIF4E-BP1 was also checked; all the treatments, including PP242 albeit to a lesser extent, reduced the mTOR kinase activity. Phosphorylation of eIF2α was also evaluated along with the relative quantification of the total protein. The inhibitory phosphorylation was particularly evident for the hypoxia treatment, but also for Torin1 and Rapamycin, while it was less clear for the treatment with Everolimus and virtually absent upon PP242 treatment. Hence, the long term treatment with Torin1 and Rapamycin and in particular the 16 hours of culture in hypoxia led also to inactivation of eIF2α, further reducing the potential for cap-dependent translation. Surprisingly we observed an apparent reduction in total eIF2α protein levels. However, this was not linked to an induction of apoptosis by the treatments (Figure S3).

p16^INK4a^ and p53 mRNA levels were not affected by the treatment with mTOR inhibitors, while hypoxia strongly reduced their mRNA relative amounts (Figure 4B). Hence the increase in p16^INK4a^ protein level (Figure 4A) cannot be ascribed to enhanced transcription or mRNA stability. The same conclusion cannot be drawn for c-MYC because all the treatments led to an increase (1.5 to 2 fold) in its mRNA level (Figure 4B).

### YBX1 can target the p16^INK4a^ 5′UTR

**Figure 5:**
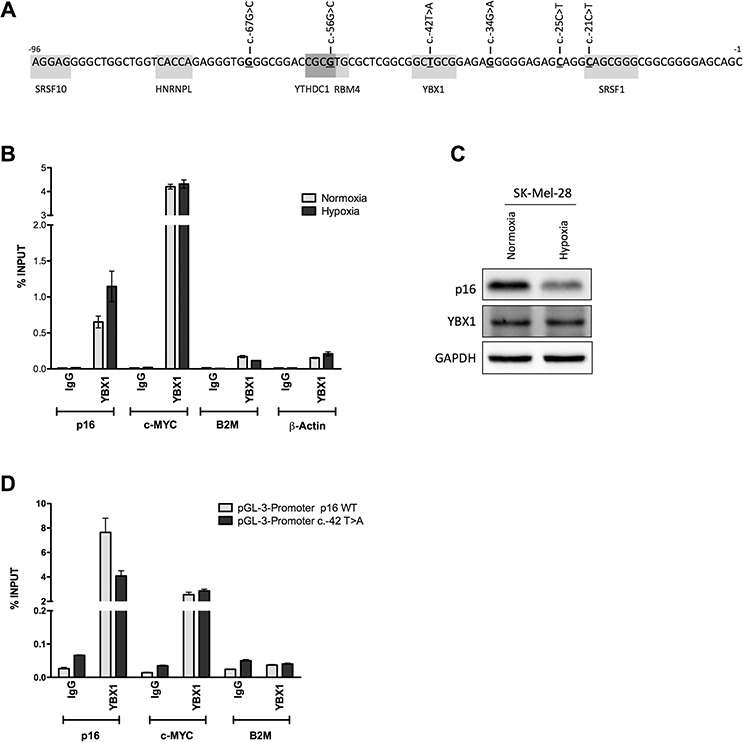
YBX1 can bind the wild type p16^INK4a^ 5′UTR but not the c.-42T>A sequence variant **A.** In-silico prediction of RNA-Binding Proteins (RBPs) targeting the proximal 96nt of the p16^INK4a^ 5′UTR. The position where nucleotide variants were previously identified in familial melanoma patients are underlined and the sequence changes are indicated. Gray boxes mark the predicted binding sites of the different RBPs. **B.** RNA Immuno-Precipitation (RIP) in SK-Mel-28 cells that express endogenous p16^INK4a^ using an antibody for the YBX1 RNA binding protein. Cells were grown in normoxia or severe hypoxia. A fraction of total RNA lysate (Input, IN) and IgG were used as positive and negative controls, respectively. Data is presented as percentage of YBX1 enrichment towards the indicated targets relative to Input RNA. A positive control (c-MYC) and two negative controls (B2M and β-Actin) were included. **C.** p16 and YBX1 protein levels were evaluated in SK-Mel-28 cells. **D.** YBX1 RIP experiment in the p16 null MCF7 cells transfected with pGL3 promoter plasmids containing either the wild type or the c.-42T>A p16^INK4a^ sequence. c-MYC and B2M were probed as positive and negative controls.

Fifteen putative binding sites for 11 different RNA binding proteins were predicted in the region spanning from the −96 position to the starting AUG a region of the p16^INK4a^ 5′UTR where most p16^INK4a^ 5′UTR sequence variants have been reported in melanoma patients [28] (Figure 5A) (see Supplementary Information). SRSF10 (FUSIP), HNRNPL, YTHDC1 (YT521-B), RBM4, YBX1 and SRSF1 (SF2/ASF) were tested by RNA immunoprecipitation (RIP) [39]. (data not shown) Only YBX1 appeared to be able to bind the p16^INK4a^ mRNA and was investigated in more details. c-MYC mRNA was used as positive control for YBX1 binding, while B2M and β-Actin were amplified as negative controls. Isotype-matched IgG were used as IP-control. Results indicated that YBX1 can bind both the p16 and c-MYC mRNAs both in SK-MEL-28 (Figure 5B). Only the binding to p16 mRNA appeared to be enhanced under hypoxia. No appreciable changes in YBX1 protein levels were observed (Figure 5C). Given the position of the predicted YBX1 binding site in the p16 5′UTR (Figure 5A), we obtained a lymphoblastoid cell line expressing the c.-42T>A p16 allele to examine if the variant can impact YBX1 binding. A p16 wild type lymphoblastoid cell line was used for comparison (Figure S4). In both cell lines c-MYC and p16 mRNAs were slightly reduced by hypoxia. Unexpectedly, YBX1 protein migration was aberrant in the p16 variant cell line (Figure S4B). While we confirmed YBX1 binding to the p16^INK4a^ 5′UTR by RIP only in p16 wild type lymphoblastoid cells (Figure S4C and not shown), the YBX1 western blot results, discouraged us from considering the results obtained with the c.-42T>A variant cells. Hence, we performed a proof-of-principle RIP assay transfecting pGL3 promoter vectors containing either the wild type or the c.-42T>A variant in the p16 null MCF7 cells (Figure 5C). Results confirmed YBX1 binding to the wild type 5′UTR and a reduction for the variant. c-MYC and B2M were used as positive and negative controls, respectively.

### The c.-42T>A p16^INK4a^ 5′UTR sequence variant results in local flexibility changes

**Figure 6:**
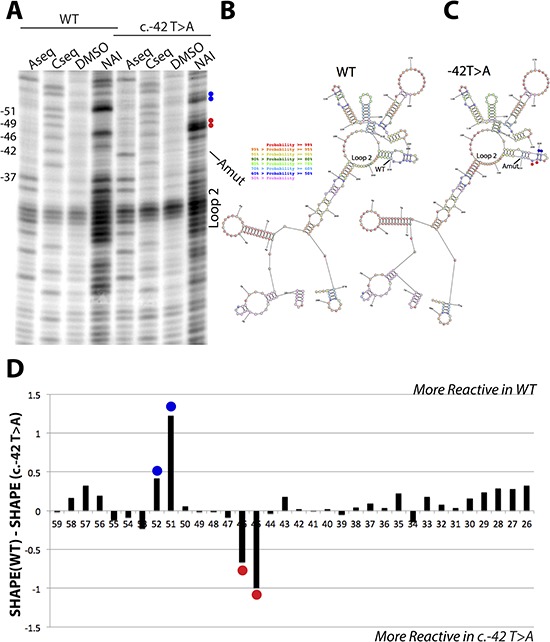
SHAPE analysis of wild type and c.-42T>A p16 5′UTR RNA structures **A.** Reverse transcription denaturing gel of wild type and c.-42T>A p16^INK4a^ 5′UTR RNA structure probing. Blue and red dots match the SHAPE differences observed in panel D. **B.** Predicted RNA secondary structure for the wild type RNA, using SHAPE restraints in the program RNA Structure. **C.** Predicted RNA secondary structure for the c.-42T>A RNA, using SHAPE restraints in the program RNA Structure. Coloring of nucleotides is denoted by probability. Blue and red dots match the SHAPE differences observed in panel D. **D.** Differential SHAPE profile between wild type and c.-42T>A p16^INK4a^ 5′UTR RNA structure profiles. Note: the nucleotides are numbered counting backwards from the AUG start codon.

To study the possible structural impact of the c.-42T>A variant on RNA structure we amplified a portion of the 5′-UTR (−288 to +3). We probed the structure using Selective Hydroxyl Acylation (SHAPE) [40]. SHAPE measures the flexibility of the RNA backbone and can be used to accurately measure RNA structure [41]. We used the recently reported SHAPE electrophile NAI [42] and we compared the structure profiles between the wild type p16^INK4a^ 5′UTR and the c.-42T>A variant using *in vitro* transcribed RNA products. Overall, the structure probing showed good agreement with the predicted RNA structure profiles, using nearest neighbor parameters for prediction [43] (Figure 6A–C). We observed strong structure stops at loops surrounding the variant site and further corroborated the presence of the adenosine mutation in the reverse transcription sequencing lane (Figure 6A, first vs fifth lane). When we compared the SHAPE profiles of the two constructs we observed flexibility differences at nucleotides 5-prime of the variant site (Figure 6D) and mapped those differences to the predicted secondary structure (Figure 6B, 6C). These differences, while overall not changing the RNA reactivity towards the SHAPE electrophile, did induce local flexibility changes. These changes may lead to an alteration in protein binding at this site. Further experiments are underway to characterize the structural changes and protein-binding impact in more detail.

### YBX1 positively controls the IRES activity of the wild type, full-length p16^INK4a^ 5′UTR

**Figure 7:**
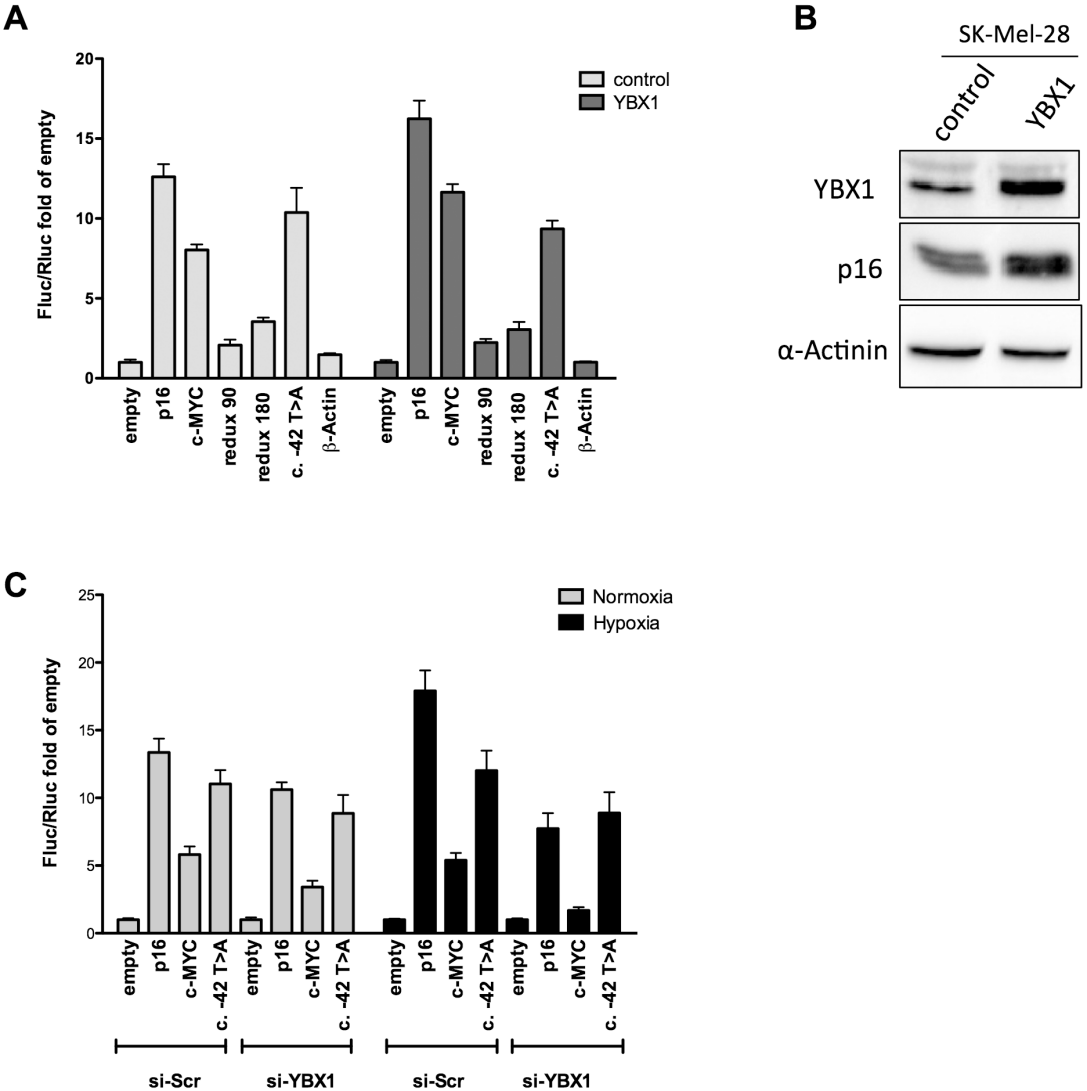
YBX1 controls p16^INK4a^ IRES activity in the bicistronic reporter vector **A.** pRuF-derived reporter constructs were transiently transfected in MCF7 cells along with an expression plasmid harboring the YBX1 cDNA (pCMV6-based) and dual luciferase assays were performed and analyzed as described previously. A pCMV6-empty vector was used as negative control. **B.** YBX1 overexpression is associated with higher endogenous p16 protein levels in SK-Mel-28 cells. Western blot of endogenous YBX1 and p16^INK4a^ proteins upon YBX1 ectopic expression in SK-Mel-28 cells. α-Actinin was used as reference. **C.** MCF7 cells were transiently transfected with a small interfering RNA directed against YBX1. 48 hours after the silencing the cells were transfected with the pRuF-derived reporter constructs. 10 hours after transfection, cells were cultured in hypoxia for additional 8 hours and dual luciferase assays were conducted as in panel A.

We then evaluated the functional impact of YBX1 protein on p16^INK4a^ 5′UTR-mediated translation efficiency. First, we over-expressed YBX1 and performed a gene reporter assay in MCF7 cells co-transfected with the panel of pRuF reporter vectors, including the one bearing the c.-42T>A variant. Ectopic expression of YBX1 led to an increase in Firefly luciferase activity with the wild type p16^INK4a^ 5′UTR pRuF plasmid, while the reporter activity was not affected with the vectors containing the two 5′UTR deletion constructs, the c.-42T>A variant and the β-Actin negative control, demonstrating a positive effect of YBX1 on p16 IRES-dependent regulation (Figure 7A). We observed an increase in luciferase activity also with the c-MYC construct, which was expected, as it has been already demonstrated that YBX1 is able to bind and positively regulate c-MYC IRES-dependent translation [44, 45]. The individual Firefly and Renilla reporter's values are presented in Figure S5A. Moreover, ectopic expression of YBX1 in the SK-Mel-28 melanoma cell line was able to enhance the endogenous p16 protein level (Figure 7B).

The knock-down of YBX1 in pRuF transfected MCF7 cells abolished the increase in Firefly luciferase activity observed with the wild type p16^INK4a^ 5′UTR in hypoxic condition (Figure 7C). The individual Firefly and Renilla reporters' values are presented in Figure S5B. Interestingly, the c.-42T>A was not associated with higher Fluc activity in hypoxia nor was affected by YBX1 silencing. The reduction in Firefly luciferase activity upon YBX1 silencing was even more evident for the c-MYC pRuF vector both in normoxia and hypoxia. YBX1 knock-down was confirmed by Western blot (Figure S5C).

## DISCUSSION

### p16^INK4a^ mRNA translation can be regulated in cis through a cellular IRES

The mechanism of action and the existence of cellular IRES is somewhat controversial [46, 47], given the lack of a precise understanding of structure/function relationships of 5′UTR sequence features that can enable or modulate ribosome loading. While bicistronic reporter vectors have been routinely used to establish the IRES potential of sequences, we developed several controls to support our results. These include: deletion constructs; qPCR assays to probe the potential for alternative transcription start sites at the p16^INK4a^ 5′UTR and control for overall transcription levels of the bicistronic mRNA, and RT-PCR assays to exclude cryptic donor or acceptor splice sites that could potentially enable cap-dependent translation of the internal Firefly reporter open reading frame. We also constructed bicistronic vectors containing IRES negative control 5′UTRs and took advantage of information on p16^INK4a^ sequence variants identified in multiple melanoma patients. Furthermore, cellular perturbation assays were employed to examine the relative activity of the different bicistronic reporter vectors, including the treatment with different inhibitors of the mTOR pathway, which are expected to selectively reduce cap-dependent translation and hypoxic stress, another condition known to exacerbate the activity of IRESs [4, 12, 48]. Overall, our results consistently indicate that the wild type 271 nt human p16^INK4a^ 5′UTR should be classified as a *bona fide* cellular IRES. Our attempts to map the IRES function to a specific sub-region of the 5′UTR were not successful, as both the proximal 90 nt and distal 180 nt seemed to be critical for this activity.

We also investigated p16 mRNA and protein levels from the endogenous *CDKN2A* locus using a melanoma-derived cell line that retains p16 expression, using polysomal profiling [28] or Western blot. c-MYC and mutant p53 are also expressed in this cell line, and as their mRNAs contain established cellular IRESs, we included the relative quantification of these two proteins as controls. qPCR was also performed on p16, c-MYC and p53 genes to correlate changes in steady-state transcript levels with those seen at the protein level. Although the western blot approach is indirect and might also be affected by changes in protein stability, the results were consistent with the potential for the p16 mRNA to be translated in cap-independent manner. For example, p16 protein levels increased upon addition of Torin1, Rapamycin and even more so with Everolimus, treatments that clearly reduced the levels of phosphorylated eIF4EBP1, but did not impact on the relative levels of p16 mRNA. The exposure of SK-Mel-28 to hypoxia, led to no change or a slight reduction in p16 protein levels, a result in apparent contrast with the proposed engagement of cap-independent translation in hypoxic stress. However, p16 mRNA levels were heavily reduced (down to 30%) under the severe hypoxia of our experimental set-up; hence, taking into account the ratio between mRNA and protein, the hypothesis of cap-independent translation of p16 in hypoxia is supported. The results also lead us to propose that p53 protein levels can be maintained in hypoxia at least in part through cap-independent translation. Relative abundance of p16 mRNA among polysomal fraction also supported the enhanced translation efficiency of this mRNA in conditions of reduced global protein synthesis.

### YBX1 can target the p16^INK4a^ 5′UTR modulating p16 post-transcriptional regulation

We searched using bioinformatics tools RNA binding proteins that could potentially act as ITAFs, *i.e*. able to facilitate the ribosome loading on the 5′UTR region of target mRNAs, for the p16 5′UTR. We were encouraged in this search by the availability of patients' derived lymphoblast cell lines presenting germline p16 5′UTR sequence variants that could serve for experimental validations. Given that most of the p16 5′UTR sequence variants identified in patients are clustered in the 96 nucleotides proximal to the AUG codon, we searched for putative RBPs predicted to be able to target that region [23] (Andreotti *et al*., submitted). Six different RBPs were tested by RIP assays in SK-Mel-28 cells and YBX1 showed binding to the p16^INK4a^ 5′UTR. YBX1 has already been reported as ITAF-like protein for c-MYC [44, 45] and is considered as an oncogene and a marker of aggressiveness for some cancer types [29, 49]. This protein is known also for its ability to bind DNA and modulate transcription [50] and to repress the CDKN2A/p16^INK4a^ promoter [51, 52]. The predicted binding site in the p16 5′UTR mRNA overlapped with a c.-42T>A variant identified in a multiple melanoma patient that harbors in trans two other variants at the positions c.-25C>T and c.-180G>A.

Collectively RIP, overexpression and silencing experiments established YBX1 as a candidate RNA binding protein (RBP), positively regulating p16 protein translation. This activity would not fit with the reported oncogenic role of this protein [29, 49] and would potentially oppose the reported inhibitory effect on p16^INK4a^ transcription [51, 52]. Thus, p16^INK4a^ 5′ UTR variants may block this potential tumor suppressive function of YBX1, while maintaining its oncogenic transcriptional repression activity. Should this paradigm occur *in vivo*, the reduction in cap-independent translation potential for the c.-42T>A variant could result in lower levels of the p16 tumor suppressor protein and favor oncogenic transformation, specifically when cancer cells activate an IRES-dependent translation program to evade environmental stress such as during hypoxia [48]. The different results in the functional and RIP assays obtained with wild type and the c.-42T>A variant motivated us to investigate the 5′UTR structure by Selective Hydroxyl Acylation (SHAPE) assay [42]. The reactivity profiles indicate local flexibility changes mapped to a predicted stem loop secondary structure that overlaps with the YBX1 RNA binding site motif (CUGCG; −43 through −39 of the p16 5′UTR) [53–55].

### Crosstalk between p53 and p16: translational regulation at the G1/S checkpoints

Recently, it was discovered that p53 can repress the expression of Fibrillarin and thereby repress cap-independent translation [34]. Our results with the HCT116 and MCF7 cell line clones indicate that p16^INK4a^ 5′UTR can be affected by p53 status and by the associated changes in relative Fibrillarin expression. Furthermore, p53 can repress the mTOR pathway by activating SESN1 and SESN2 [56]. Notably, YBX1 translation has been recently found to be reduced upon mTOR inhibition [38]. In addition, an interaction between miR-34a, a p53 target miR [57], and YBX1 3′UTR was reported [58]. In fact, we have shown that activation of p53 leads to YBX1 protein decrease both through the mTOR pathway inhibition and miR-34a activation [37]. These findings along with the results of the present study would imply that p53 can indirectly control p16 protein levels via YBX1, but the ultimate consequences on p16 protein levels may vary depending on the relative effect of YBX1 on p16^INK4a^ transcription and translation.

Our study is the first demonstration of a mechanism regulating mRNA translation efficiency of p16^INK4a^ that might be relevant for the maintenance of its critical role in cellular checkpoint and senescence, particularly in stressful conditions that impair protein synthesis. Impaired p16 mRNA translation, for example under hypoxia, could provide a mechanistic clue to explain melanomagenesis associated with p16^INK4a^ 5′UTR germline sequence variants.

## MATERIALS AND METHODS

### Cell lines, culture and treatment procedures

Breast adenocarcinoma-derived MCF7 cells and melanoma-derived SK-Mel-28 cells were obtained from InterLab Cell Line Collection (ICLC, Azienda Ospedaliera Universitaria San Martino-IST, Genoa, Italy). MCF7vector and MCF7shp53 cells were obtained from the Agami lab [36]. HCT116 p53^+/+^ and p53^−/−^ were obtained from the Vogelstein lab (The Johns Hopkins Kimmel Cancer Center, Baltimore, USA). All cell lines were maintained in standard media (see Supplementary Information). To selectively inhibit cap-dependent translation MCF7 or SK-Mel-28 cells were treated with different mTOR inhibitors Rapamycin (Sigma Aldrich, Milan, Italy), Everolimus (Sigma Aldrich), PP242 (Sigma Aldrich) and Torin1 (Aurogene, Rome, Italy) using a 50nM dose for 16 hours or a 250nM dose for 2 hours. For hypoxic conditions, we cultured MCF7 or SK-Mel-28 using a GENbox plastic jar with a GENbag hypoxia generator (Biomerieux, Florence, Italy) for 16 hours.

### Plasmid construction, dual luciferase assays, protein extraction and western blot

pRuF bicistronic and pGL3-derived vectors containing the entire p16^INK4a^ 5′UTR were generated as previously described [28] and as outlined in Supplementary Information. Luciferase assays were performed as previously described [28]. MCF7 and SK-Mel-28 cells were treated with mTOR inhibitors (250nM for 2 hours or 50nM for 16 hours) or grown in hypoxia for 16 hours. Cells were then harvested, washed once with PBS and soluble proteins were extracted from pellets using RIPA lysis buffer supplemented with Protease Inhibitor cocktail (Roche, Milan, Italy) and with Phosphatase Inhibitor cocktail (Sigma-Aldrich). Protein extracts were quantified and SDS-PAGE electrophoresis performed as previously described [59]. The list of antibodies is provided in Supplementary Information. To analyze the effect of YBX1 over-expression on endogenous p16^INK4a^ protein, pCMV6-empty or pCMV6-YBX1 were transiently transfected in SK-Mel-28 cells with LT-2020 reagent (Mirus, Tema Ricerca, Milan, Italy).

### RNA extraction, qPCR and Ribonucleoprotein ImmunoPrecipitation (RIP) assays

MCF7 cells were seeded into 6-well plates and transfected at 70–80% confluence with 2 μg of the pRuF reporter vectors containing the wild type or the deleted p16^INK4a^ 5′UTR. Alternatively, SK-Mel-28 cells were treated with 50nM mTOR inhibitors or grown in hypoxia. Twenty-four hours after transfection or 16 hours after the different treatments, cells were harvested and processed as previously described (34). RIP was performed as previously described [39] (see Supplementary Information)

### RNA interference

YBX1 protein levels were reduced in MCF7 cells using 25nM of specific small interfering RNA si-RNA (IDT, Coralville, IA, USA) transiently transfected using Interferin transfection agent (PolyPlus, EuroClone, Milan, Italy) in 24-well plate format. Forty-eight hours after silencing, cells were further transfected with different pRuF reporter vectors (-empty, - p16^INK4a^ wild type, -c-MYC and - p16^INK4a^ c.-42T>A) using Fugene HD transfection reagent (Promega). Eight hours post-transfection cells were cultured respectively in normoxic or hypoxic condition for additional 16 hours. Then, after a total of 72 hours from silencing, cells were washed and lysed using PLB1X and gene reporter assay was performed as described above. The same protein extracts were also used for western blot experiments where the YBX1 (59-Q, Santa Cruz) protein levels and GAPDH (6C5, Santa Cruz) were detected.

### Sucrose gradient fractionation of cytoplasmic cell lysates and polysomal profiling

RNA associated with polysomes or present in the cytoplasm in light fractions (co-sedimenting with individual ribosomal subunits or with 80S) were obtained using 10%-50% sucrose-gradient fractionation and UVC-coupled fraction collection following the protocol described in [28] and in Supplementary Information.

### Construction of RNA sequences for RNA structure probing

Inserts were amplified from pRuF plasmid DNA using the following primers:

T7 forward: TAATACGACTCACTATAGGGCCC AACCTGGGGCGA

Reverse: CATGGCATATGATCGATGCTGCT

Primers produce dsDNA from −288nt to +3nt in the 5′-UTR/Translation start site boundary. Procedures for *In Vitro* Transcription and Acylation of RNA, Reverse Transcription of modified RNA and characterization of reverse transcription stops are described in Supplementary Information.

## SUPPLEMENTARY MATERIALS AND METHODS, FIGURES


