# Supplementary material for: The 5′-untranslated region of p16INK4a melanoma tumor suppressor acts as a cellular IRES, controlling mRNA translation under hypoxia through YBX1 binding
Source: Oncotarget. 2015 Oct 17;6(37):39980–94. doi: 10.18632/oncotarget.5387 (PMC4741874; doi:10.18632/oncotarget.5387)
Supplement: Supplementary file 1 [file oncotarget-06-39980-s001.pdf]

SUPPLEMENTARY MATERIALS AND METHODS

Cell lines and culture conditions

Cells were maintained either in RPMI or DMEM (MCF7 and SK-Mel-28) supplemented with 10% Fetal Bovine Serum (FBS), 2 mM L-Glutamine, Pen/Strep mixture and, when appropriate, Non Essential Amino acids (Gibco, Life Technologies, Milan, Italy) in humidified atmosphere at 37°C and 5% CO_2. In the case of the MCF7vector and MCF7shp53 cells, medium was also supplemented with 0.5 µg/ml Puromycin to maintain the selection for the construct expressing the short hairpin targeting p53. Lymphoblastoid cells derived from two melanoma patients, one wild type and the second heterozygous for the c.–42T>A $p16^{INK4a}$ 5′UTR variant were transformed by EBV and the established cell lines stored at the Galliera Genetic Bank Repository (Genoa, Italy). EBV-transformed lymphoblastoid cells were cultured using RPMI medium supplemented with 10% FBS and antibiotics. Cells were routinely checked to exclude the presence of mycoplasm.

Bicistronic reporter plasmid

pRuF bicistronic vector containing the whole $p16^{INK4a}$ 5′UTR was generated as previously described [1]. Exploiting EcoRI and NdeI restriction sites within pRuF-empty backbone and PCR primer tails, $p16^{INK4a}$ 5′UTR fragments were cloned using T4 Ligase enzyme (New England BioLabs, EuroClone, Milan, Italy) and *XL1Blue* bacterial cells. As positive control, the c-MYC 5′UTR region containing the previously identified IRES site was PCR amplified from MCF7 RNA using the primers pair described in previous studies [2] modified to include tails containing the EcoRI and NdeI restriction sites, and cloned in pRuF reporter vector as described above. With a similar approach two negative controls were developed inserting the 5′UTR of β-globin and β-actin [3]. As an additional negative control, the reverse complement $p16^{INK4a}$ 5′UTR sequence (named p16INV) was cloned in pRuF plasmid as negative control and we also generated two deletion constructs, denominated Redux 180 and Redux 90, where the proximal 90 nucleotides and the distal 180 nucleotides of the $p16^{INK4a}$ 5′UTR were deleted respectively. The correct insertions of the desired fragments were confirmed by EcoRI and NdeI endonucleases double digestion and direct sequencing (BMR Genomics, Padua, Italy). pCMV6-Entry Myc-DDK tagged vector to overexpress YBX1 was purchased from Origene (Tema Ricerca, Bologna, Italy). Monocistronic vectors based on the pGL3-promoter plasmid, containing the c-MYC, p16 wild-type or p16 c.-42T>A variant 5′UTR upstream of the Firefly luciferase were constructed as described in [1]. The pRL-SV40 plasmid was used as a control for transfection together with the monocistronic constructs. Transfection grade plasmids were obtained using the Pure Yield Midiprep system according to the manufacturer's instructions (Promega, Milan, Italy).

Dual luciferase assays

Luciferase assays with the cloned pRuF vectors were performed in 24-well plates with MCF7, HCT116, MCF7vector or MCF7shp53 cells, as previously described [1]. Briefly, cells were transiently transfected with Fugene HD transfection reagent (Promega) and 400 ng of pRuF constructs or 350 ng of pGL3-promoter + 50 ng of pRL-SV40 plasmids. When appropriate, 100ng of the pCMV6-empty or pCMV6-YBX1 expression vector were co-transfected along with the different pRuF reporter vectors. Twenty-four hours after transfection cells where lysed with Passive Lysis Buffer (PLB) 1X and Firefly and Renilla luciferase activity was measured with the Infinite M200 multi-plate reader (Tecan, Milan, Italy). In the experiments that needed cap-dependent inhibition, 8 hours after transfection MCF7 cells were treated with 50 nM mTOR inhibitors or cultured in hypoxia for additional 16 hours prior to the luciferase assay.

Protein extraction and western blot

SDS-PAGE was performed as previously described [4], proteins were transferred on nitrocellulose membranes using the semi-dry i-Blot transfer system (Invitrogen, Life Technologies) and specific protein levels detected using primary antibodies diluted in 1% non-fat skim milk in PBS-T. The endogenous levels of phospho- and total eIF4E-BP1 (ab27792, AbCam, Milan, Italy and R-113, Santa Cruz Biotechnology, Milan, Italy, respectively) were analyzed in MCF7 cells. For SK-Mel-28 cells the endogenous expression of p16 (N-20, Santa Cruz), p53 (DO-1, Santa Cruz), c-MYC (9E10, Santa Cruz), HIF1α (610959, BD Biosciences, Milan, Italy), PCNA (F-2, Santa Cruz), ERK1 (C-16, Santa Cruz), phospho- and total eIF4E-BP1, phospho- and total-eIF2α (AbCam) was tested. GlycerAldehyde 3-Phosphate DeHydrogenase (GAPDH -6C5, Santa Cruz) and α-Actinin (H-2, Santa Cruz) were used as reference protein. To detect the immune-reactive bands we used the ECL select reagent (Amersham, GE-Health Care, Milan, Italy) and the ChemiDoc XRS+ documentation system with the ImageLab software (BioRad, Milan, Italy).

High-content imaging analysis of global protein translation

To investigate the impact of treatments with mTOR inhibitors or hypoxia on global protein sysntesis, SK-Mel-28 cells were exposed to 50 nM Rapamycin or 50 nM Torin1 or cultured in hypoxic chamber for a total of 16 hours. 3 hours prior to sample analysis, the culture medium was removed and replaced with fresh, methionine free medium supplemented with an amino acid analog of methionine containing an alkyne moiety, following the procedure of the Click-iT® HPG Alexa Fluor® 488 Protein Synthesis Assay Kit (Molecular Probes, Invitrogen, Life Technologies). Relative quantification of protein synthesis was performed by acquiring images by the PerkinElmer Operetta® High Content Imaging System, using DAPI staining to visualize nuclei. Images were analyzed and quantified using Columbus software.

RNA extraction and qPCR on total RNA

To extract RNA, cells were washed once with phosphate buffer saline (PBS). Total RNA was extracted using the RNAeasy mini Kit (Qiagen, Milan, Italy) according to the manufacturer's instructions. In-column DNAse treatment (Qiagen) was performed to remove DNA contamination during total RNA extraction. Purity and concentration of RNA was evaluated using a NanoDrop ND1000 spectrophotometer. cDNA was generated starting from 1 µg of RNA by using the RevertAid™ First Strand cDNA Synthesis Kit (ThermoFisher, Milan, Italy). Quantitative Real-time PCR (q-PCR) was performed using a cDNA aliquot equivalent to 25 ng of converted RNA using a CFX96 qPCR Thermal cycler (BioRad, Milan, Italy) and the 2X KAPA SYBRGreen FAST qPCR Master Mix (Kapa Biosystems, Resnova, Ancona, Italy). In the transiently transfected MCF7 cells the regions spanning the Renilla and the Firefly luciferase mRNA and the portion in between the two reporter genes were amplified using specific primers (Eurofins MWG Operon, Ebersberg, Germany – all primers' sequences utilized in this work are available upon request). The relative mRNA abundance was quantified as explained in a previous study [1]. Endogenous p16^{INK4a}, c-MYC and p53 relative mRNA levels were measured from SK-Mel-28 treated cells. The relative quantitation of endogenous mRNAs from treated SK-Mel-28 was obtained using the comparative Ct method (ΔΔCt), taking into account the efficiency of cDNA synthesis by the quantification of the glyceraldehyde 3-phosphate dehydrogenase (GAPDH) and β-2microglobulin (B2M) reference genes.

RNA extraction and qPCR on subpolysomal and polysomal RNA fractions

The protocol for sucrose gradient fractionation was previously described [1]. Fractions corresponding to subpolysomal peaks were pooled together and the same was done for peaks corresponding to polysomal fractions (see Figure 3B for example of profiles). Prior to RNA extraction by organic solvent and salt/ethanol precipitation 15 ng of spike-in Luciferase control RNA (Promega) was added to both pools. RNA was resuspended in DEPC water, quantified at nanodrop and processed for qPCR analysis as described above. The sequences of all primers were taken from previous publications or designed using primer blast and are available upon request.

Predicting RNA binding protein targeting the p16^{INK4a} 5′UTR

The RNA-Binding Protein (RBPDB) [5] and SpliceAid [6] databases were used in order to predict which RNA binding proteins (RBPs) could bind to the 5′UTR of p16^{INK4a} mRNA. RBPDB and SpliceAid predict the binding of RBPs to a desired sequence using data assembled from experimentally observed RNA-binding sites for RBPs and splicing factors, manually curated from the literature. 22 different RBPs (EIF4B, ELAVL1, FUS, KHDRBS3, KHSRP, MBNL1, NONO, RBM4, RBMX, SFRS1, SFRS13A, SFRS9, Vts1, YBX1, YTHDC1, hnRNPH1, hnRNPH2, hnRNPF, hnRNPL, SC35, SRp40, SRp30c) were predicted to bind to the full-length p16^{INK4a} 5′UTR sequence. Fifteen putative binding sites for 11 different proteins were found in the region spanning from the -96 position to the starting AUG. We focused on this region where most p16^{INK4a} 5′UTR sequence variants have been reported in melanoma patients [1]. RNA immunoprecipitation (RIP) protocol [7] was performed using the SK-MEL-28 cell line and, since the IRES function of the p16^{INK4a} 5′UTR was apparently stimulated in hypoxia, culturing cells both in normoxia or hypoxia.

Ribonucleoprotein ImmunoPrecipitation (RIP) assays

RIP was performed as previously described [7]. Briefly, 12 x 10^6 cells were lysed in 500 µl of NT2 buffer (50 mM Tris-HCl pH7.7, 150 mM NaCl, 1 mM MgCl2; 0.05%NP40, 1U/ul RNase IN, 20 mM DTT, 1% BSA, Protease inhibitor cocktail) pre-chilled at 4°C and syringed using an U100 insulin needle. Lysates were centrifuged at 10000g for 10 minutes then the supernatant was pre-cleared by interaction with protein-A-coated agarose beads (equilibrated in NT2 buffer) overnight

at 4°C in constant shaking (100 µl slurry beads / 500 µl lysate). 150 µl of the pre-cleared lysate were mixed with protein G coated agarose beads with a specific primary antibody (e.g. YBX1, Ab 59-Q, Santa Cruz) (or control IgG) conjugated for 6 hours at 4°C then washed twice in NT2 buffer. 20 µl Protein-G-coated slurry agarose beads were conjugated with 4 µg antibody at room temperature for 2 hours, washed and equilibrated in NT2 lysis buffer before use. RNA was isolated from the different samples (immunoprecipitated anti-YBX1, IgG and pre-cleared input) by TriZol, as recommended by the manufacturer, retrotranscribed into cDNA by RevertAid™ First Strand cDNA Synthesis Kit (ThermoFisher) and used as template for PCR or for a q-PCR analysis that was conducted as described above.

In Vitro Transcription and Acylation of RNA

RNA was transcribed from amplified inserts using T7 Megascript kit from Ambion, following manufacturer's protocol. In a typical *in vitro* modification protocol, RNA was heated in metal-free water for two minutes at 95°C. The RNA was then flash-cooled on ice. The RNA 3X SHAPE buffer (333 mM HEPES, pH 8.0, 20 mM $MgCl_2$, 333 mM NaCl) was added and the RNA was allowed to equilibrate at 37°C for ten minutes. To this mixture, 1 µL of 10X 2-methylnicotinic acid imidazolide electrophile (NAI) stock in DMSO or DMSO only was added. The reaction was permitted to continue until the desired time. Reactions were extracted once with acid phenol:chloroform (pH 4.5 ± 0.2) and twice with chloroform. RNA was precipitated with 40 µL of 3M sodium acetate buffer (pH 5.2) and 1 µL of glycogen (20 µg/µL). Pellets were washed twice with 70% ethanol and resuspended in 10 µL RNase-free water.

Reverse Transcription of modified RNA

^{32}P-end-labeled DNA primer (reverse primer above) was annealed to 3 µg of total RNA by incubating at 95˚C for two minutes followed by a step-down cooling (2 deg/sec) to 4˚C. To the reaction, first-strand buffer, DTT and dNTPs were added, pre-incubated at 52 ˚C for one minute, followed by Superscript III (2units/µL final concentration) addition. Extensions were performed for ten minutes. To the reaction, 1 µL of 4M NaOH was added and allowed to react for 5 minutes at 95°C. 10 µL of Gel Loading Buffer II (Ambion, Inc.) was then added, and cDNA extensions were resolved on 8% denaturing (7 M Urea) polyacrylamide gels (29:1 acrylamide:bisacrylamide, 1X TBE). Sequencing lanes are from DMSO control treated cells.

Characterization of reverse transcription stops

cDNA extensions were visualized by phosphorimaging (STORM, Molecular Dynamics). cDNA bands were integrated with SAFA [8] SHAPE reactivities were normalized to a scale spanning 0 to 1.5, where 1.0 is defined as the mean intensity of highly reactive nucleotides [9]. RNA secondary structures were predicted using RNA structure software, incorporating the SHAPE structure probing as a restraint [10].

SUPPLEMENTARY REFERENCES

1. Bisio A, et al. Functional analysis of CDKN2A/p16INK4a 5′-UTR variants predisposing to melanoma. Hum Mol Genet. 2010; 19:1479–91.
2. Stoneley M, et al. C-Myc 5′ untranslated region contains an internal ribosome entry segment. Oncogene. 1998; 16:423–8.
3. Andreev D.E, et al. Differential contribution of the m7G-cap to the 5′ end-dependent translation initiation of mammalian mRNAs. Nucleic Acids Res. 2009; 37:6135–47.
4. Bisio A, et al. Identification of new p53 target microRNAs by bioinformatics and functional analysis. BMC Cancer. 2013; 13:552.
5. Cook K.B, et al. RBPDB: a database of RNA-binding specificities. Nucleic Acids Research. 2011; 39:D301–D308.
6. Piva F, et al. SpliceAid: a database of experimental RNA target motifs bound by splicing proteins in humans. Bioinformatics. 2009; 25:1211–3.
7. Latorre E, et al. Downregulation of HuR as a new mechanism of doxorubicin resistance in breast cancer cells. Mol Cancer. 2012; 11:13.
8. Das R, et al. SAFA: semi-automated footprinting analysis software for high-throughput quantification of nucleic acid footprinting experiments. RNA. 2005; 11:344–54.
9. Gherghe C, et al. Definition of a high-affinity Gag recognition structure mediating packaging of a retroviral RNA genome. Proc Natl Acad Sci U S A. 2010; 107:19248–53.
10. Hajdin C.E, et al. Accurate SHAPE-directed RNA secondary structure modeling, including pseudoknots. Proc Natl Acad Sci U S A. 2013; 110:5498–503.

SUPPLEMENTARY FIGURES

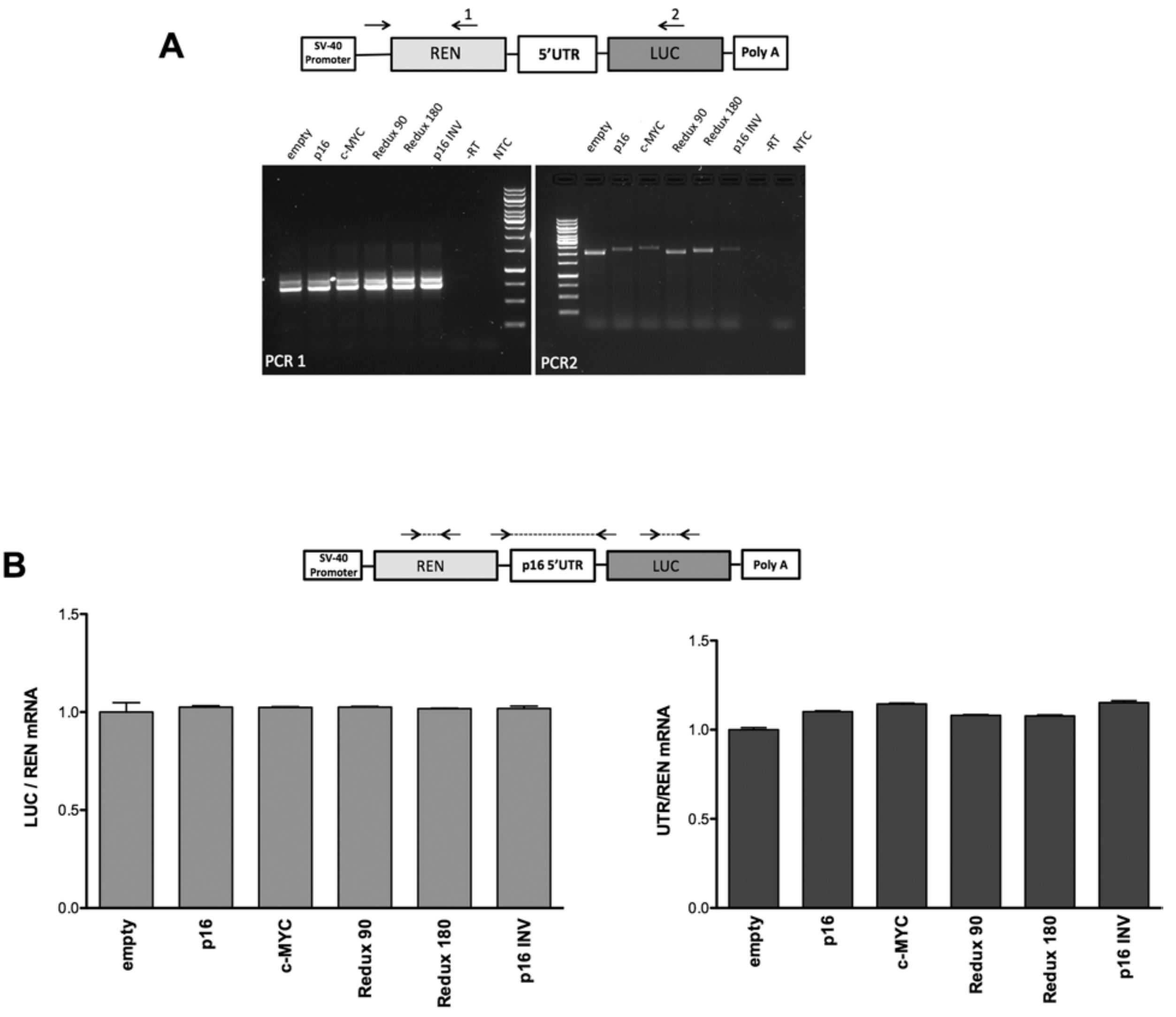

Supplementary Figure S1: The p16^{INK4a} 5′UTR embedded in a bicistronic vector does not alter splicing nor act as cryptic promoter. To exclude the possibility that the observed increases in Fluc activity (Figure 1) were due to the presence of an alternative splicing event, we used an RT-PCR approach on total RNA extracted from transiently transfected MCF7 cells with the different pRuF reporter vectors (**panel A**). The Forward Primer was chosen to anneal at the region immediately downstream the transcriptional start site but upstream the small intron present in pRL-SV40, while Reverse Primer 1 anneals to the Renilla (REN) and Reverse Primer 2 anneals to the Firefly (LUC) cDNA sequence, as schematically depicted in the top panel; the forward primer was coupled with either reverse primer. The Firefly Reverse primer may enable detection of alternative splicing events that could lead to cap-dependent translation of this reporter. The results of the two PCRs are shown as electrophoretic run images in the left (PCR 1) and right (PCR 2) panels, respectively. Amplicons of the expected size were obtained, indicating that both reporters were part of a single transcript. The two bands visible in the left panel are un-processed and processed transcripts, respectively, as a chimeric intron is included immediately downstream of the pSV-40 promoter in the pRLSV-40 vector backbone, on which the pRuF plasmid is based. A vector containing the inverted p16^{INK4a} 5′UTR sequence was used as a control, since promoter, unlike enhancers, are considered directional sequence elements. The approximate position of the PCR primers is indicated in the schematic representation of the pRuF vector.

Higher Fluc activity could be due to the production of an additional transcript containing only the Fluc reporter, resulting from a cryptic promoter within p16^{INK4a} 5′UTR. Therefore, we performed qPCR experiments to measure the relative amounts of Rluc, Fluc and 5′UTR mRNA regions (**panels B**). Three different segments of the bicistronic mRNA were amplified and quantified separately: one inside the Renilla (REN) luciferase sequence; the second inside the Firefly (LUC); and the third encompassing the 5′UTR sequence. The position of the three primer pairs is schematically depicted. Results were plotted as ratios between Fluc / Rluc and 5′UTR / Rluc mRNAs (left and right panels, respectively). Bars plot averages and standard deviations of at least three independent biological replicates. No significant differences were observed among the different amplicons and using the different pRuF constructs indicated below the bar graph, and described in the main text and Figure 1, demonstrating the absence of a cryptic promoter.

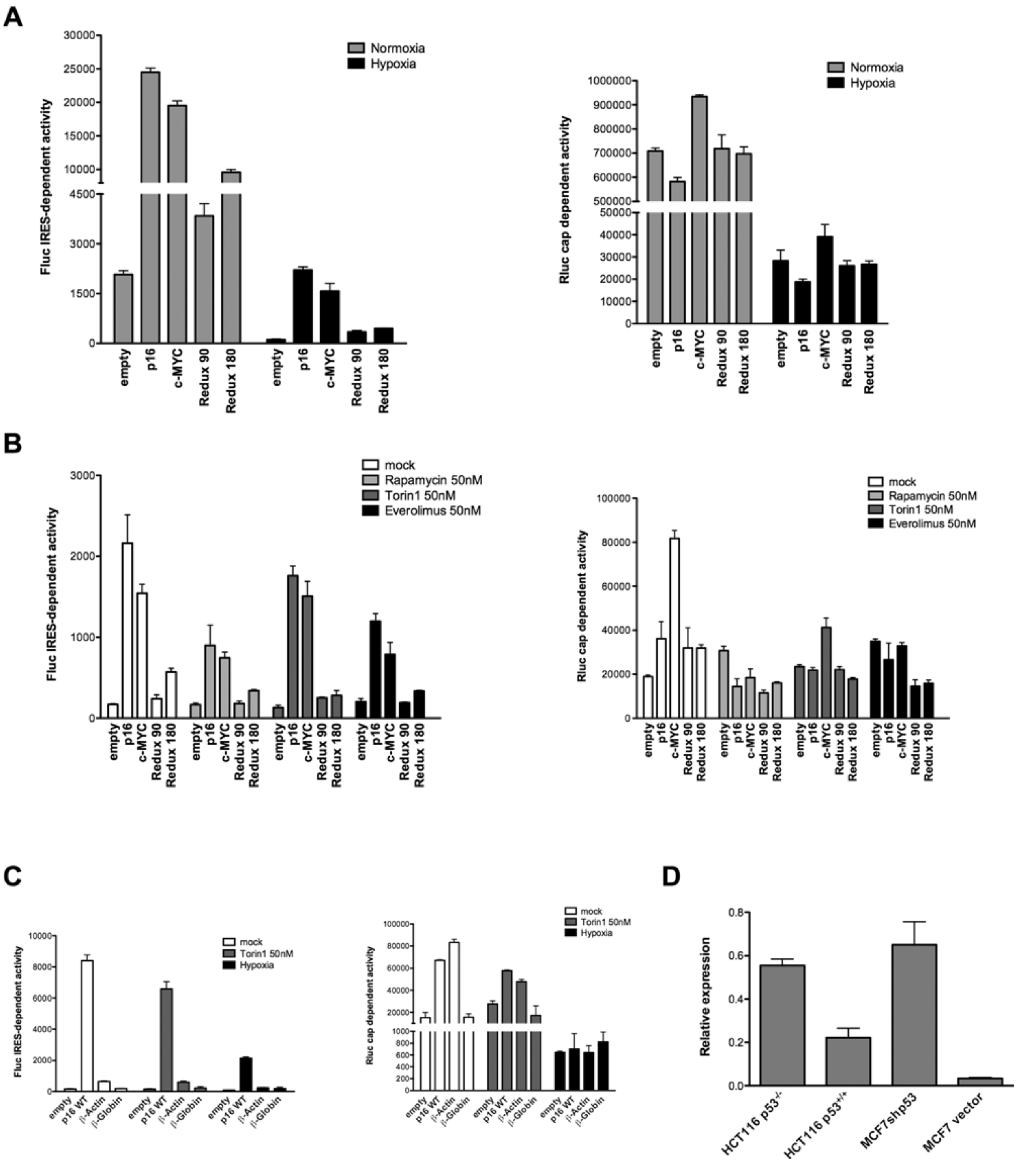

Supplementary Figure S2: Supporting data for Figure 2. A–C. Results deriving from gene reporter assays shown in Figure 2A 2B and 2D left panel are plotted as Fluc (left histograms) and Rluc (right histograms) raw data respectively in panel A, B and C. **D.** The endogenous Fibrillarin mRNA levels were measured by qPCR in the two cancer-derived cell line pairs differing for p53 status used in Figure 2E and 2F. B2M and GAPDH mRNA levels were used as reference genes and presented as ΔCt values.

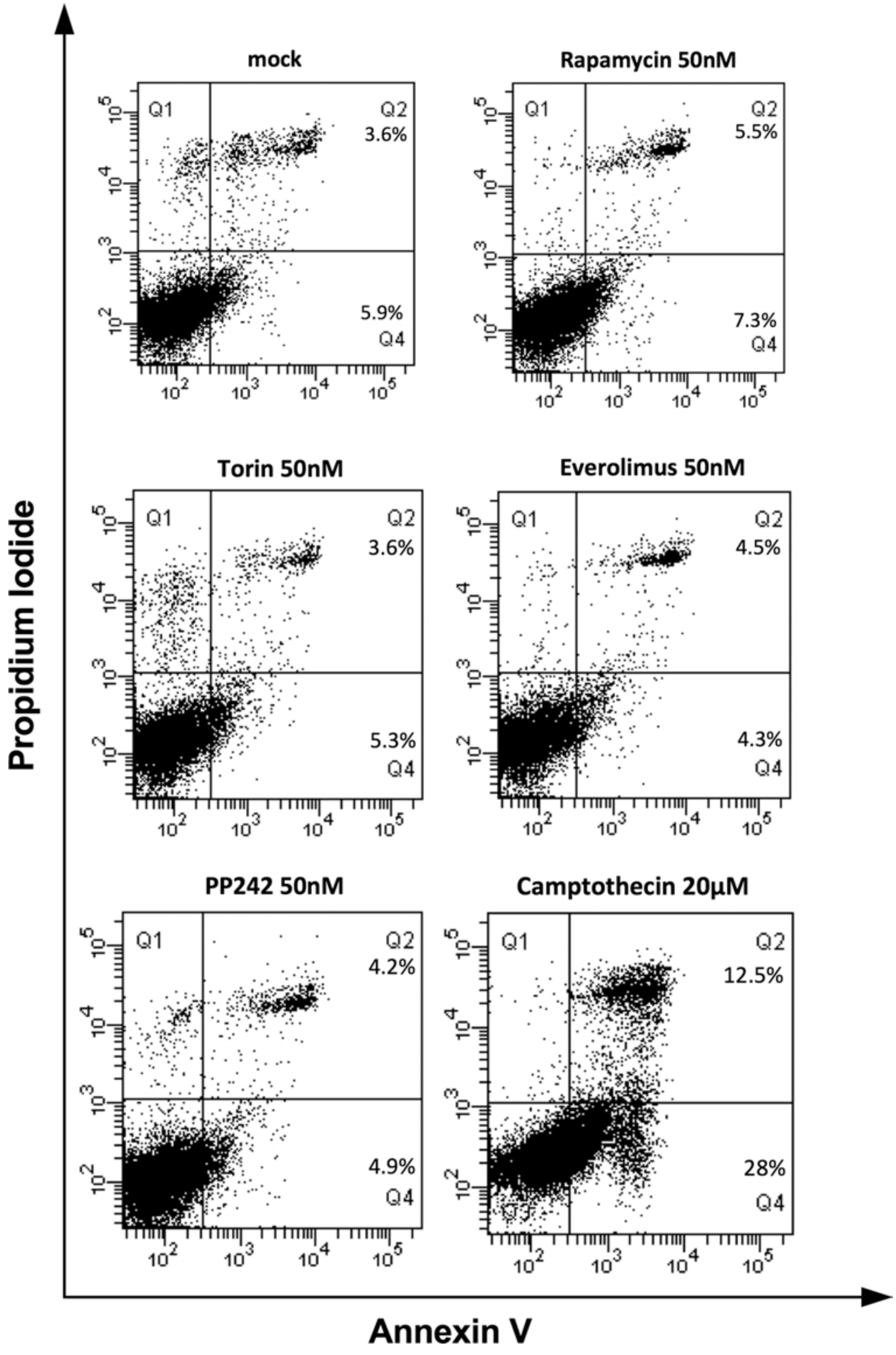

Supplementary Figure S3: mTOR inhibitors as well as hypoxia did not induce apoptosis in SK-Mel-28 cells. SK-Mel-28 cells were seeded in 10-cm dishes (Corning, Euroclone, Milan, Italy); after 24 h, cells were treated with Rapamycin, Torin, Everolimus, PP242 (all at 50nM) and 20 µM Camptothecin (as positive control) or cultured in hypoxia for 16 hours. In order to have more information about cells viability, we recovered and analyzed also cells that were in suspension after the treatments. The FITC AnnexinV Apoptosis Detection kit I (BD Pharmingen, Milan, Italy) was used for the staining following the manufacturer's protocol. Propidium Iodide was used as a nucleic acid dye (BD Pharmigen). Flow cytometry analysis was conducted using a FACSCanto II instrument (BD Biosciences). Percentages of early apoptotic cells (Q4 quadrant) and late apoptotic cells (Q2 quadrant) are shown.

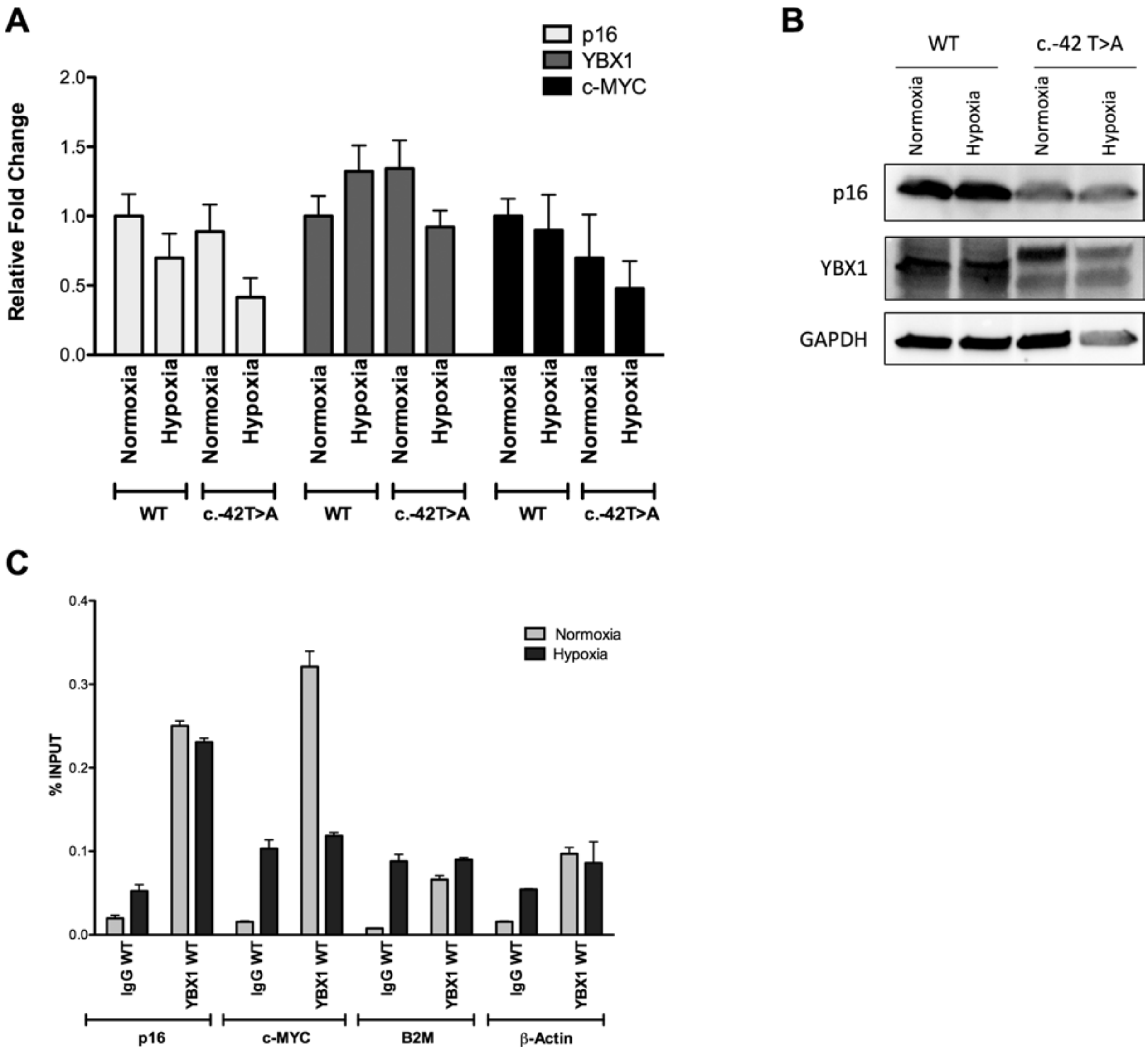

Supplementary Figure S4: qPCR, western blot and RIP assays in lymphoblastoid cells. A. p16, YBX1 and c-MYC mRNA levels were evaluated in two lymphoblastoid cell lines (wild type and c.-42T>A p16 sequence variant that lies within the predicted YBX1 binding region: -43-CUGCG) grown in normoxic and hypoxic conditions. B2M and GAPDH served as reference genes. **B.** p16 and YBX1 protein levels evaluated by western blot in the same pair of lymphoblastoid cells. Unexpectedly, the migration of YBX1 was aberrant in the c.-42T>A cells. This finding did not make us confident in using the p16 variant cell model to evaluate the impact of the T>A change on YBX1 binding to the p16^{INK4a} 5′UTR. **C.** YBX1 binding on p16^{INK4a} 5′UTR was instead tested using the p16^{INK4a} wild type lymphoblastoid cells cultured in normoxia and hypoxia by RIP assays. The experiment was performed as for Figure 5B. YBX1 exhibited binding to the p16^{INK4a} 5′UTR both in normoxia and hypoxia, while the binding to c-MYC appeared to be restricted to the normoxia condition.

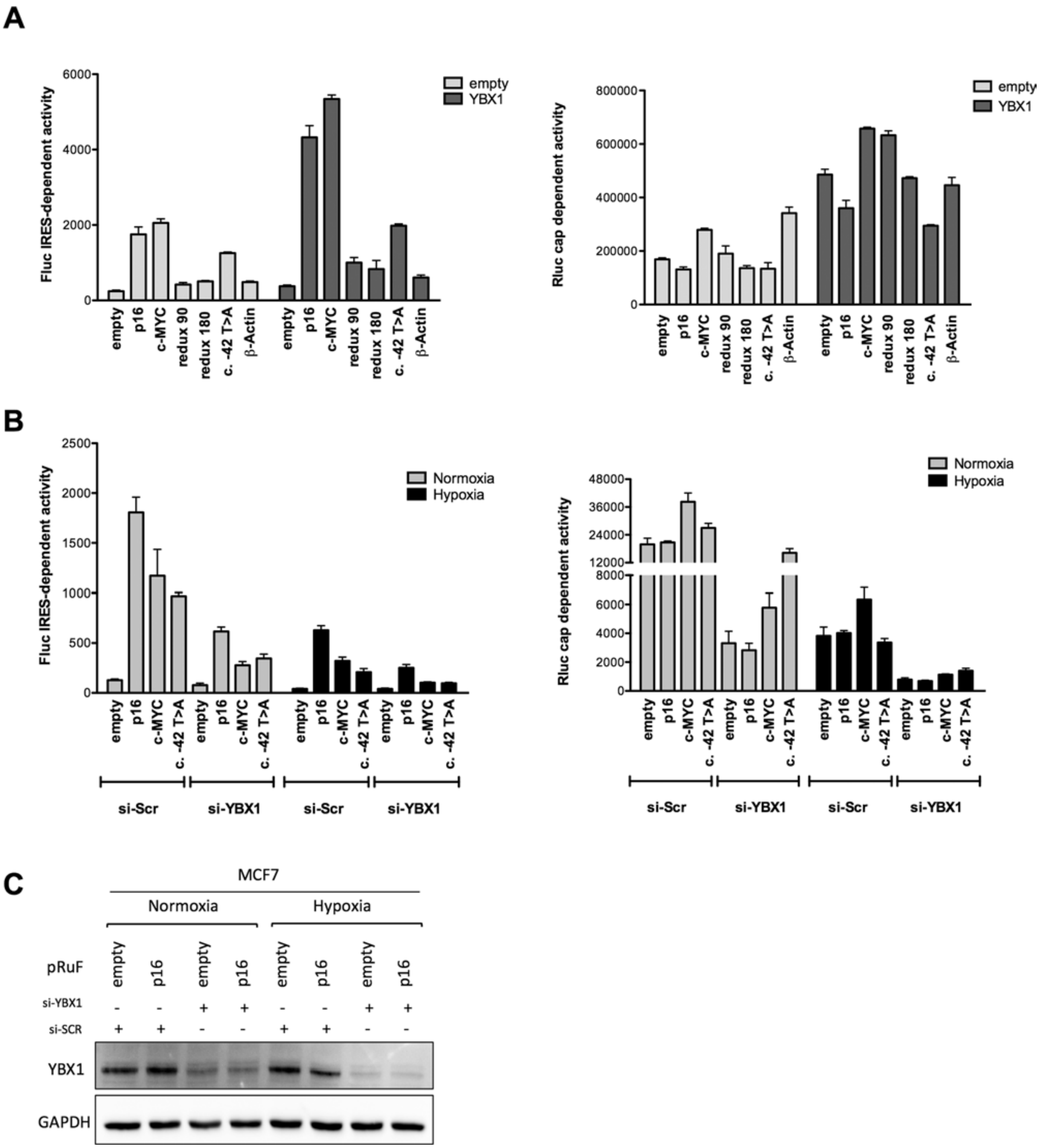

Supplementary Figure S5: Luciferase assays and western blot in MCF7 cells, supporting data in Figure 7. A, B. Results deriving from gene reporter assays shown in Figure 7A and 7C were plotted as Fluc (left histograms) and Rluc (right histograms) raw data, respectively in panel A and B. YBX1 overexpression led to higher Renilla activity also for the empty vector control, possibly due to an effect on the transcription levels of the viral promoter in the vector. Conversely, YBX1 silencing led to lower Renilla activity, also for the empty vector control. **C.** MCF7 lysates from dual luciferase assays were used for western blot analysis to evaluate the effective reduction in YBX1 protein level after silencing with siRNA. Extracts both from cells transfected with empty and wild type p16 5′UTR pRuF vectors were loaded. GAPDH protein served as loading control.
